# Electroacupuncture versus exercise in patients with knee osteoarthritis: Study protocol for a randomized controlled trial

**DOI:** 10.1371/journal.pone.0305105

**Published:** 2024-06-11

**Authors:** Xue-Zhou Wang, Rui-Kang Wang, Qiang Liu, Guang-Xia Shi, Bao-Hong Mi, Cun-Zhi Liu, Jian-Feng Tu, Jian-Hao Lin

**Affiliations:** 1 International Acupuncture and Moxibustion Innovation Institute, School of Acupuncture-Moxibustion and Tuina, Beijing University of Chinese Medicine, Beijing, China; 2 Arthritis Clinic and Research Center, Peking University People’s Hospital, Beijing, China; 3 Arthritis Institute, Peking University, Beijing, China; 4 Beijing University of Chinese Medicine Third Affiliated Hospital, Beijing, China; Universitat de Valencia, SPAIN

## Abstract

**Purpose:**

Knee osteoarthritis (KOA) is a common disorder among middle and older individuals. Electroacupuncture and exercise are present as two popular physical therapies for the management of KOA, and both were demonstrated to produce considerable results. However, the clinical decision-making process between these therapeutic interventions remains challenging due to the limited evidence of distinctions in their respective effects. This study aims to evaluate the clinical effect and cost effectiveness of electroacupuncture versus exercise in patients with KOA.

**Study design and methods:**

This is a randomized controlled trial in which 196 symptomatic KOA patients will be randomly assigned 1:1 either to the electroacupuncture group (n = 98) and the exercise group (n = 98). Patients in the electroacupuncture group will receive acupuncture with electric stimulation 3 times a week for 8 weeks, whereas patients in the exercise group will receive neuromuscular training twice a week for 8 weeks. Education concerning KOA management will be provided in both therapies. Co-primary outcomes include changes in numerical rating scale (NRS) and Knee injury and Osteoarthritis Outcome Score (KOOS) Activities of Daily Living (ADL) subscale from baseline at week 8. Secondary outcomes include KOOS Pain subscale, KOOS knee-related Quality of Life (QOL) subscale, Short Form 6 Dimensions (SF-6D), five-level EuroQol five-dimensional questionnaire (EQ-5D-5L), Credibility/ Expectancy Questionnaire, Patient’s global assessment (PGA), 30-second Chair Stand Test (30s-CST), 40m (4*10m) Fast Paced Walk Test (40m FPWT), and Daily Physical Activity level (DPA).

**Discussion:**

The results of this study will provide evidence regarding differences between these 2 physical therapies in multiple aspects and will provide specific guidance for the development of treatments based on the needs of individual patients.

**Trial registration:**

ChiCTR2300070376.

## Introduction

Knee osteoarthritis (KOA) is an important cause of chronic pain and physical dysfunction among middle-aged and elderly populations [1]. Recent years seen a rise in the worldwide prevalence of KOA [2], with a prevalence of symptomatic KOA reached 8.1% in China [3]. For the rapid aging of population, the social burden of KOA will increase further. Although non-steroidal anti-inflammatory drugs (NSAIDs) remain the first-line of treatment, their limited benefits and potential for gastrointestinal bleeding and cardiovascular events were noted [4]. Consequently, nonpharmacologic therapies are receiving increased attention for the management of mild to moderate KOA [5–7].

Intervention for the management of KOA is varied. Exercise showed strong evidence in managing KOA, with multiple forms of exercise delivery proven to be better than non-exercise [8–11]. Acupuncture is another nonpharmacologic therapy which showed favorable evidence. Compared to the sham acupuncture, acupuncture can relieve pain and dysfunction [12–14]. Considering the safety and potential benefits, acupuncture was partly recommended by certain guidelines [6,7,15]. Electroacupuncture is the type of acupuncture recommended for research by National Institute for Health and Clinical Excellence. Its potential better effect was observed in our previous study [13]. Benefits to the convenience and standardization of operation, electroacupuncture is more widely used in clinical practice. In early studies focused on both acupuncture and exercise, acupuncture was designed to be a supplement [16–18]. Considering the cost of time and expenses, it can be difficult for patients to adopt multiple nonpharmacologic therapies. The popularity of acupuncture and exercise varies across different regions. Understanding their respective advantages and characteristics can provide a basis for personalized selection. For lack of attention, evidence regarding the differences between acupuncture and exercise is insufficient. To our knowledge, only one trial compared acupuncture with exercise directly [19]. However, the inclusion of patients in surgery waiting list was limited in generalization. From an economic point of view, both acupuncture and exercise were proved to be cost-effective options for KOA in the developed countries. In a German study compared acupuncture with routine care in treating KOA, the overall Incremental Cost-Effectiveness Ratio (ICER) was 17,845 Euros per Quality-Adjusted Life Year (QALY) gained [20]. In another Denmark study compared supervised exercise with education, the ICER ranged from 6,229 to 20,688 Euros per QALY [21]. However, few studies focused on the difference between the cost-effectiveness of acupuncture and exercise directly. Meanwhile, due to economic and medical resource constraints in developing countries, the cost-effectiveness of acupuncture and exercise may be disparate from that in developed countries. To save conserve resources, it is necessary to find the more cost-effective therapy.

This randomized controlled trial aims to: (1) evaluate the effectiveness of electroacupuncture versus exercise in alleviating pain and improving function in patients with KOA; (2) compare the cost effectiveness of acupuncture and exercise in treating KOA.

## Methods

### Study design

**Fig 1:**
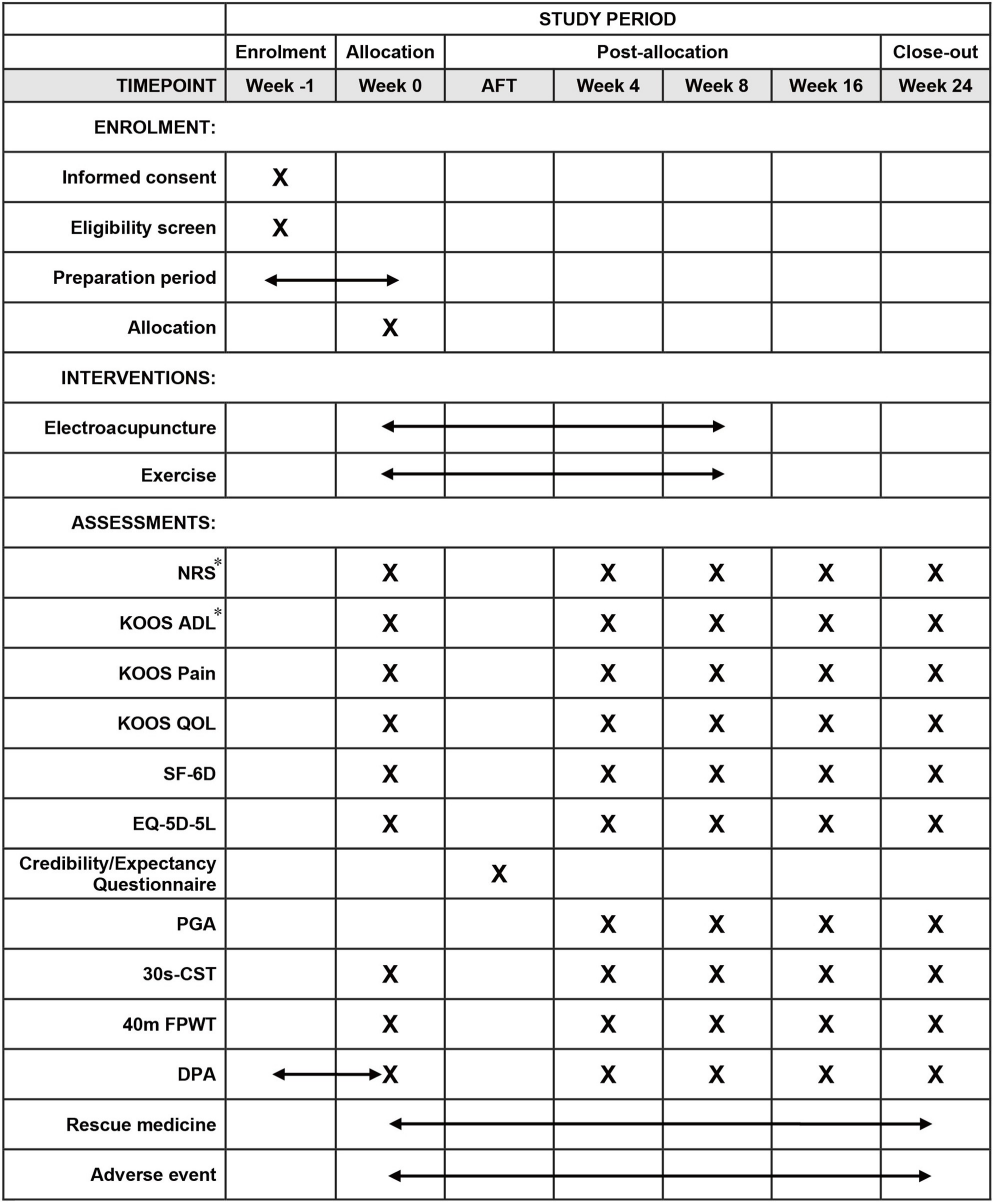
SPIRIT schedule. *, primary outcome. Abbreviation: AFT, after the first treatment; KOOS, Knee injury and Osteoarthritis Outcome Score; ADL, Activities of Daily Living; QOL, Quality of Life; SF-6D, Short Form 6 Dimensions; EQ-5D-5L, Five-level EuroQol five-dimensional questionnaire; PGA, Patient’s global assessment; 30s-CST, 30-second Chair Stand Test; 40m FPWT, 40m (4x10m) Fast Paced Walk Test; DPA, Daily Physical Activity level.

This will be a pragmatic, two-arm, randomized controlled trial conducted in 2 centers in China. A total of 196 KOA patients will be randomly assigned to the electroacupuncture group and the exercise group in a ratio of 1:1. The trial duration will be 24 weeks, comprising 8 weeks of treatment and 16 weeks of follow up. The study protocol has been approved by The Ethics Committee of Dongzhimen Hospital Affiliated to Beijing University of Chinese Medicine (2022DZMEC-165-02) and Peking University People’s Hospital (2021PHB431-001), and registered on Chinese Clinical Trial Registry (ChiCTR2300070376. Date: April 04, 2023. Version 1.0.). This protocol will be reported in accordance with Standard Protocol Items: Recommendations for Interventional Trials (SPIRIT) [22]. Fig 1 illustrates the SPIRIT schedule. S1 File shows the SIPRIT checklist and S2 File shows the original protocol approved by the ethics committee.

### Study site

Recruitment, intervention and follow up will be conducted at Peking University People’s Hospital and Dongzhimen Hospital Affiliated to Beijing University of Chinese Medicine simultaneously. Following randomization, patients will be allotted to the respective group. Recruitment will take place on outpatient departments, with intervention and follow up occurring in dedicated rooms within the hospitals.

### Participants

#### Inclusion criteria

Patients will be eligible if they: (1) aged 45–75, any gender; (2) meet the American College of Rheumatology (ACR) diagnostic criteria [23] of KOA; (3) report knee pain for more than 3 month; (4) had radiological examination within 6 months indicating Ⅱ or Ⅲ in Kellgren–Lawrence (KL) grade [24] with medial more than lateral tibiofemoral osteoarthritis on at least one knee; (5) score 4 or greater in numerical rating scale (NRS) for the past week; (6) are willing to sign the informed consent. Diagnosis and KL classification will be performed by orthopedic surgeons.

#### Exclusion criteria

Patients will be ineligible if meet any of the following points: (1) prior knee surgery on the evaluating knee or pending any surgery for either knee; (2) knee pain due to other pathologies (e.g., meniscus tear, rheumatoid arthritis, joint cavity infection, malignancy, gout, lumbosacral diseases with symptom of lower extremity, etc.); (3) arthroscopy history within 1 year or intraarticular injection history within 6 months for the evaluating knee; (4) either knee having received acupuncture/exercise therapy in the last 6 months; (5) severe acute or chronic organic or neuropsychiatric disorder; (6) disorders of coagulopathy (e.g., hemophilia); (7) pacemakers or epilepsy; (8) pregnancy preparation, pregnant or lactation period; (9) participated in other clinical studies within the past 1 month.

### Allocation

Patients will be randomly assigned to the electroacupuncture group or exercise group in a ratio of 1:1, using block randomization. Random sequences will be programmed by an independent statistician using STATA 17 software. Concealed allocation will be maintained through the use of opaque sealed envelopes containing serial numbers on the outside and a sheet of paper with the random sequence on the inside. Following obtain the written informed consent (S3 File), eligible patients will be asked to complete a general information form including name, gender, age, medical history, etc. as the baseline demographic assessment. Due to the particularity of Daily Physical Activity level (DPA) which cannot be evaluated immediately at the time of recruitment, patients will enter a 1-week preparation period after screening. When the preparation period is finished, the remaining baseline assessments will be measured. After that, a researcher will sequentially open an envelope and allocate the patient to the corresponding center for intervention. The random allocation sequence and the opaque sealed envelopes will be kept separately by a researcher.

### Blinding

Outcome evaluators and statistical analysts will be blinded. Due to the considerable differences between the two therapies, blinding of therapists and patients is not feasible.

### Interventions

For patients with unilateral knee osteoarthritis, treatments will be performed on the affected side which will be defined as the evaluating side. For those with bilateral osteoarthritis, treatments will be performed on both knees, with the more serious side being evaluated. In the event of similar conditions on both sides, flipping a coin to decide which side to evaluate. For both groups, the compliance of the intervention will be recorded. Any other treatment that may affect symptoms during the trial is prohibited, including drug therapy (NSAIDs, opioids, etc.) and physical therapy (tuina, hydrotherapy, etc.). When necessary, patients can receive Diclofenac Sodium Enteric-coated Tablets (Voltaren, Beijing Novartis Pharma Co., Ltd) as the rescue medication, except within 48 hours prior to the evaluation; timing and dosage will be documented meticulously. Education will be accompanied by both interventions, in a form of simulating doctor-patient communication in real treatment environment. This will include information regarding the definition, clinical manifestation, diagnosis, etiology, risk factors, anatomy of the knee joint, treatment, prevention measures and pain manage of KOA, with the aim of helping patients to better understand the condition. In addition, patients with a Body Mass Index (BMI) >25 will be encouraged to lose weight.

#### Electroacupuncture

Patients in the electroacupuncture group will receive treatment 3 times a week, which has been confirmed by our previous study [25] as the better frequency for 8 weeks (a total 24 sessions). An interval of one or two days between each session will be advisable, and repeated session in one day will be prohibited.

A semi-standardized acupoints selection method will be used. The prescription was formulated based on clinical experience and reviews, and is consistent with our previous study [13].

The semi-standardized prescription contains fixed and optional acupoints. *Dubi* (ST35), *Neixiyan* (EX-LE5), *Ququan* (LR8), *Xiyangguan* (GB33) and an *Ashi* point (the point where the patient feels the most pain) are fixed points in this study, which means they will be chosen for all patients. An additional 22 acupoints make up the optional acupoints. The acupuncturist will select three of these optional acupoints based on the meridians corresponding to the pain site. Further details of electroacupuncture are provided in Table 1, with acupoint locations, corresponding needle type and depth of insertion shown in Table 2. Positioning of all acupoints follows World Health Organization’s Standard Acupuncture Locations. The electroacupuncture intervention will be delivered by three acupuncturists from Dongzhimen Hospital Affiliated to Beijing University of Chinese Medicine, each possessing over three years of clinical experience.

**Table 1:** Details of the electroacupuncture intervention.

Item	Parameter
Needling instrument	length: 25–50 mm, diameter: 0.25 mm; Hwato, Suzhou, China
Retaining time	30min
Treatment sessions	24
Frequency	Three times a week
Manipulation	After the needle inserted, lift, thrust and thrill smoothly for at least 10s to achieve De qi.
Electrical acupoints	Wire 1: LR8 and GB33. Wire 2: 2 of 3 adjunct acupoints.
Electric parameter	Disperse-dense wave. The current will be increased slowly from zero to the degree which the participant can feel but tolerate.

De qi is a multidimensional sensation of numbness, soreness, distention, heaviness during acupuncture stimulation.

**Table 2:** Acupoints’ locations, corresponding needle type and depth of insertion.

Acupoint	Name	Location	Needle type	Depth of insertion
Fixed acupoints	Dubi (ST35)	On the anterior aspect of the knee, in the depression lateral to the patellar ligament	0.25×40mm	25~40mm
	Neixiyan (EX-LE5)	On the anterior aspect of the knee, in the depression medial to the patellar ligament	0.25×40mm	25~40mm
	Ququan (LR8)	On the medial aspect of the knee, in the depression medial to the tendons of the semitendinosus and the semimembranosus muscles, at the medial end of the popliteal crease	0.25×25mm	20~25mm
	Xiyangguan (GB33)	On the lateral aspect of the knee, in the depression between the biceps femoris tendon and the iliotibial band, posterior and proximal to the lateral epicondyle of the femur	0.25×40mm	25~40mm
	Ashi point	The point where the patient feels most pain	depends on the location	
Optional acupoints for yangming meridian syndrome	Futu (ST32)	On the anterolateral aspect of the thigh, on the line connecting the lateral end of the base of the patella with the anterior superior iliac spine, 6 cun^a^ superior to the base of the patella	0.25×40mm	25~40mm
	Liangqiu (ST34)	On the anterolateral aspect of the thigh, between the vastus lateralis muscle and the lateral border of the rectus femoris tendon, 2 cun superior to the base of the patella	0.25×40mm	25~40mm
	Heding (EX-LE2)	On the anterior aspect of the thigh, in the depression superior to the base of the patella	0.25×25mm	13~20mm
	Zusanli (ST36)	3 cun directly below ST35, and one finger-breadth lateral to the anterior border of the tibia	0.25×50mm	25~50mm
	Fenglong (ST40)	On the anterolateral aspect of the leg, lateral border of the tibialis anterior muscle, 8 cun superior to the prominence of the lateral malleolus	0.25×40mm	25~40mm
Optional acupoints for three-yin meridian syndrome	Xuehai (SP10)	On the anteromedial aspect of the thigh, on the bulge of the vastus medialis muscle, 2 cun superior to the medial end of the base of the patella	0.25×40mm	25~40mm
	Yingu (KI10)	On the posteromedial aspect of the knee, just lateral to the semitendinosus tendon, in the popliteal crease	0.25×40mm	25~40mm
	Yinlingquan (SP9)	On the tibial aspect of the leg, in the depression between the inferior border of the medial condyle of the tibia and the medial border of the tibia	0.25×50mm	25~50mm
	Xiguan (LR7)	On the tibial aspect of the leg, inferior to the medial condyle of the tibia, 1 cun posterior to SP9	0.25×40mm	25~40mm
	Sanyinjiao (SP6)	On the tibial aspect of the leg, posterior to the medial border of the tibia, 3 cun superior to the prominence of the medial malleolus	0.25×40mm	25~40mm
	Taixi (KI3)	On the posteromedial aspect of the ankle, in the depression between the prominence of the medial malleolus and the calcaneal tendon	0.25×25mm	13~25mm
	Taichong (LR3)	In the depression anterior to the junction of the first and second metatarsal bones	0.25×25mm	13~25mm
	Gongsun (SP4)	On the medial aspect of the foot, anteroinferior to the base of the first metatarsal bone, at the border between the red and white flesh	0.25×25mm	13~25mm
Optional acupoints for taiyang meridian syndrome	Weiyang (BL39)	On the posterolateral aspect of the knee, just medial to the biceps femoris tendon in the popliteal crease	0.25×40mm	25~40mm
	Weizhong (BL40)	On the posterior aspect of the knee, at the midpoint of the popliteal crease	0.25×40mm	25~40mm
	Chengshan (BL57)	On the posterior aspect of the leg, at the connecting point of the calcaneal tendon with the two muscle bellies of the gastrocnemius muscle	0.25×50mm	25~50mm
	Kunlun (BL60)	On the posterolateral aspect of the ankle, in the depression between the prominence of the lateral malleolus and the calcaneal tendon	0.25×25mm	13~20mm
Optional acupoints for shaoyang meridian syndrome	Fengshi (GB31)	On the lateral aspect of the thigh, in the depression posterior to the iliotibial band where the tip of the middle finger rests, when standing up with the arms hanging alongside the thigh	0.25×50mm	25~50mm
	Yanglingquan (GB34)	On the fibular aspect of the leg, in the depression anterior and distal to the head of the fibula	0.25×40mm	25~40mm
	Waiqiu (GB36)	On the fibular aspect of the leg, anterior to the fibula, 7 cun proximal to the prominence of the lateral malleolus	0.25×40mm	25~40mm
	Xuanzhong (GB39)	On the fibular aspect of the leg, anterior to the fibula, 3 cun proximal to the prominence of the lateral malleolus	0.25×25mm	13~20mm
	Zulinqi (GB41)	On the dorsum of the foot, distal to the junction of the bases of the fourth and fifth metatarsal bones, in the depression lateral to the fifth extensor digitorum longus tendon	0.25×25mm	8~13mm

^a^ 1 cun (≈20 mm) is defined as the width of the interphalangeal joint of patient’s thumb.

#### Exercise

The intervention of the exercise group will be consistent with the NEuroMuscular Exercise program (NEMEX) in Good Life with Osteoarthritis in Denmark (GLA:D®) [26]. GLA:D® contains education and neuromuscular training for patients and has been found to yield favorable outcomes in various countries [27]. In order to compare the benefits of electroacupuncture with the exercise itself, the NEMEX program rather than the entire GLA:D® program was adopted. The exercise program consists of 16 NEMEX sessions, to be held twice weekly over an 8-week period, with each session lasting approximately 60 minutes. Each session is divided into three parts: warm-up, circuit exercise program, and cool-down (Table 3).

**Table 3:** Details of the exercise intervention.

Part	Detail		Time
Warming up	Ergometer cycling		10 minutes
Circuit program	Core stability/postural function	A. Pelvic-lift B. Sit-ups	40 minutes
	Postural orientation	A. Slide-exercise forward-backward B. Slide-exercise sideways	
	Lower extremity muscle strength	A. Hip abductors/hip adductors B. Knee extensors/knee flexors	
	Functional exercises	A. Chair stands B. Stair climbing	
Cooling down	Walking, mobility exercises and stretching exercises		10 minutes

Warm-up: This part involves 10 minutes of ergometer cycling. The workload will be set individually to improve the efficiency and safety during subsequent exercise.

Circuit exercise program: This part is the nucleus of each session, comprising core stability exercise, dynamic posture control exercise, muscle strength exercise and daily functional exercise. The circuit exercise program comprises 4 segments, each consisting of 2 distinct movements. Each movement is to be repeated thrice, for a total of ten rounds. Furthermore, each movement is stratified into 3 levels, with incremental intensity for each level. The appropriate level of intensity is determined based on the patient’s ability to complete the movement and their pain feedback.

Cool-down: This part consists of walking, mobility exercises for the lower extremities and stretching exercises for the lower extremity muscles. This part is expected to last approximately 10 minutes.

The exercise program will be carried out in groups of 8–10 patients. All exercise interventions will be supervised by 3 licensed therapists from Peking University People’s Hospital, each possessing over three years of clinical experience. More details of the exercise program could be found in S4 File and published protocol [28].

### Outcomes

#### Primary outcomes

There are two primary outcomes in this trial. The primary outcome of pain will be changes in Numerical Rating Scale (NRS) and the primary outcome of function will be changes in the Knee injury and Osteoarthritis Outcome Score (KOOS) Activities of Daily Living (ADL) [29] from baseline at week 8. NRS is an 11-point pain assessment scale ranging from ‘no pain’ (0) to ‘worst pain’ (10). KOOS is an extension of the Western Ontario and McMaster University Osteoarthritis Index (WOMAC) [30], designed to evaluate knee injuries and osteoarthritis. KOOS Activities of Daily Living (ADL) subscale comprises 17 items and has a score range of 0–100, with lower scores indicating worse dysfunction.

#### Secondary outcomes

The secondary outcomes of this study include self-reported outcomes, objective outcomes of physical function, and a biological indicator outcome.

KOOS Pain [29]: A 9-item pain evaluation scale, with scores ranging from 0 to 100. Lower scores indicating greater levels of pain.

KOOS knee-related Quality of Life (QOL) [29]: A 4-item quality of life evaluation scale, with scores ranging from 0 to 100. Lower scores indicating poorer quality of life.

Short Form 6 Dimensions (SF-6D) [31]: A quality of life evaluation scale derived from the SF-36, with 6 items for estimating the cost-effectiveness. Higher scores indicating poorer quality of life.

Five-level EuroQol five-dimensional questionnaire (EQ-5D-5L) [32]: A generic preference-based quality of life evaluation scale, with 5 items for jointly estimating the cost-effectiveness. Higher score indicates poorer quality of life.

Credibility/Expectancy Questionnaire [33]: A 6-item scale for evaluating patients’ credibility/expectancy to the intervention. This scale will be measured within 5 min after the finish of first treatment.

Patient’s global assessment (PGA) [34]: A 5-point ordinal scale for evaluating the patient’s overall perception of the effect.

Thirty-second Chair Stand Test (30s-CST) [35]: A test of lower body strength, dynamic balance and sit-to-stand activity. The patient will be requested to sit in a chair with their feet flat on the floor, shoulder width apart, knees bent slightly more than 90 degrees, arms crossed at wrists and close to their chest. Then, within 30 seconds, the patient will be measured the times they could complete as many full stand-to-sit cycles as possible. The hips and knees require be fully extended when standing up; the hips require to fully touching the chair when sitting down. Additionally, the height of chairs needs to be unified.

Forty meters (4x10m) Fast Paced Walk Test (40m FPWT) [36]: A test of walking speed over short distances and changing direction during walking. Patients will be asked to walk back and forth twice on the 10-meter walkway completely as fast as possible without running. The time spent walking on the walkway will be recorded.

Daily Physical Activity level (DPA): DPA contains 1 week’s daily average steps and walking time, which will be recorded by portable pedometers (Polygon H-215G; Bestek Electronics Co., Ltd, Taiwan, China). The patient will be provided with a pedometer at the commencement of the preparatory period. The pedometer is required to be worn at all times except sleeping, bathing and water sports. At each visit, the researcher will record the data collected by the pedometer for the preceding week.

Rescue medicine: Any use of rescue medicine will be recorded, including the time and dosage.

The Credibility/Expectancy Questionnaire will be measured within a 5-minute period following the initial treatment session. PGA will be measured at week 4, 8, 16 and 24. Rescue medicine will be recorded within 24 hours after each taking. Other secondary outcomes will be assessed at baseline and at week 4, 8, 16 and 24. Additionally, the NRS and the KOOS ADL will be assessed at week 4, 16 and 24 as secondary outcomes.

### Adverse events

The safety of both treatments will be assessed by adverse events. Researchers and experts will determine whether any adverse events are related to the intervention within 24 hours of their occurrence. Serious adverse events will be referred to the principal investigator for further judgment and treatment. Common adverse events associated with electroacupuncture include pain, local haematoma, infection and dizziness. Common adverse events associated with exercise therapy include pain, muscle strain, spasm and joint swelling.

### Data management

All data will be recorded in detail on the case report forms, and any additional information or modifications need to be signed by the researcher. The completed CRF will be in the custody of two independent researchers who will input data doubly for proofreading. Any raw data will be stored for a period of five years post-study completion to enable readers to access these data by contacting the corresponding author, with the exception of patient-identifiable information.

### Sample size

Based on previous study [13,37], we estimated that the difference in effect size between the 2 groups would be no more than 0.5. Between-group variations in NRS and KOA were expected to be 1 and 8 at week 8, respectively, with the standard deviations (SD) of 2 and 16. With 2-side α = 0.025 (adjusted for two primary outcomes), 77 patients per treatment group are required to test a difference in effect size of 0.5 with 80% power. Taking into account a 20% dropout rate, a total of 196 patients are expected to be recruited.

### Statistical analysis

SPSS 26.0 software will be used for statistical processing. Measurement data will be expressed as mean and standard deviation, or median and interquartile ranges. Counting data will be expressed as percentages.

All randomly grouped patients with at least 1 session of intervention received will be included in the modified intention-to-treat (mITT) analysis. Multiple imputation will be used to fill the missing data. Five datasets for the missing data will be imputed by the observation value of age, gender, body mass index, KL grade and outcomes from other timepoints. All between-group comparison on repeated measure continuous outcomes (NRS, KOOS ADL, KOOS QOL, SF-6D, EQ-5D-5L, 30s-CST and 40m FPWT, DPA, except PGA) will be assessed by mixed-effect models using outcome scores at all assessed timepoints as the dependent variable, intervention as the main factor, and the baseline value as the covariate. PGA will be assessed by chi-squared test. Sensitivity analysis will be conducted on the primary outcomes (NRS and KOOS ADL) in 2 datasets. First, the dataset without imputation will be analyzed to address potential imputation bias. Second, the per-protocol (PP) set, which will be defined as receiving a minimum of 19 (electroacupuncture) or 12 (exercise) sessions with no obvious protocol violation, will be analyzed to address the compliance. For primary outcomes, *P* < 0.025 will be considered statistically significant (Bonferroni correction). Secondary outcomes will be considered exploratory, and *P* < 0.05 will be considered statistically significant.

### Health economic evaluation

An economic evaluation will be conducted from a societal perspective. At the conclusion of the 8-week treatment period, the costs associated with electroacupuncture or exercise therapy will be summarized. Since participants will not be permitted to undergo any other treatments during the study period, only study-related direct medical costs and direct non-medical costs will be calculated. These include registration fees, treatment fees, transportation costs, and any associated delays. Direct medical costs will encompass registration and treatment fees. The registration fee for each treatment will be estimated based on the patient’s self-reported choice of registered doctor. Treatment fees will be determined according to the real-time treatment prices provided by the National Medical Security Bureau. Non-medical costs will be derived from patient-reported values. QALYs for each patient over the study period will be computed using EQ-5D-5L and SF-6D from the multiple imputed dataset. Cost-effectiveness analysis will be conducted by calculating the ICER between the two groups. Missing data in the costs will be imputed by intra-group mean values.

### Exploratory analysis

A multiple linear regression analysis will be conducted to assess the association between patient characteristics and the effect of the two therapies. Factors such as gender, baseline pain intensity, KL grade and expectation will be included as independent variables. This part is exploratory.

### Study status

This trial is currently recruiting participants. After the trial registration completed, the first patient was recruited on May 09, 2023. Recruitment is expected to be completed in June 2025.

## Discussion

KOA has long plagued the elderly, and it’s also a significant contributor to societal burden. Patients tend to seek nonpharmacologic therapies, such as acupuncture (the most popular complementary medicine [38]) and exercise (a core component of guideline-based recommendations [6,7]). Numerous studies supported the respective benefits of acupuncture and exercise, yet tiny of them compared the differences to guide appropriate treatment for specific populations.

In this trial, frequencies of the two therapies will not be equal, which is to reflect the practical frequency in clinical practice. All intervention details were designed to closely emulate the clinical reality, with the aim of reacting real situation in clinical practice. Since the main symptoms of KOA are pain and dysfunction, both will be selected as primary outcomes separately in order to clearly elucidate the differences in symptom improvement between the two interventions. In addition to self-reported outcomes, objective functional tests will also be included, thus enabling the detection of both perceived effect and objective activity improvement. In terms of generalizability, the current 2 centers incorporating both traditional Chinese medicine hospital and modern hospital. Consequently, a broader spectrum of patients will be included. Further, the exploratory analyses will focus on providing evidence for different populations.

One limitation of the trial is that neither the therapist nor the patient can be blinded, leading to potential instability of the results. Patients’ expectations will enhance the placebo or nocebo effect, and the lack of blinding may bring bias. Nevertheless, this study was designed to compare the effectiveness rather than the efficacy, thus the nonspecific effect should not be excluded. Patient’s expectations will be assessed to help explaining the difference between the two therapies. In addition, the researchers maintained a neutral position to therapies, which can reduce the bias from the study design. Further limitation involves the sustainability of benefits. For a chronic disease like KOA, a 24-week assessment may not suffice to offer a comprehensive understanding. Definitive conclusions about the long-term effects between the 2 interventions necessitate further real-world studies with long term follow-up.

This study will verify the differences of effect between electroacupuncture and exercise for the management KOA. The results will provide evidence for specific options of nonpharmacologic therapies in the management of KOA.

## Supporting information

S1 File SPIRIT checklist. (DOC)

S2 File Original protocol approved by the ethics committee. (DOCX)

S3 File Model consent form. (DOCX)

S4 File Checklist for protocols according to the TIDieR guideline. (DOCX)
