# Supplementary material for: Electroacupuncture versus exercise in patients with knee osteoarthritis: Study protocol for a randomized controlled trial
Source: PLoS One. 2024 Jun 11;19(6):e0305105. doi: 10.1371/journal.pone.0305105 (PMC11166276; doi:10.1371/journal.pone.0305105)
Supplement: S2 File — (DOCX) [file pone.0305105.s002.docx]

**Original protocol approved by the ethics committee**

Electroacupuncture versus Exercise in Patients with Knee Osteoarthritis: Study Protocol for A Randomized Controlled Trial (The translated version)

**1. Research content**

Purpose: To evaluate the difference in efficacy between electroacupuncture and exercise therapy in the treatment of knee osteoarthritis

Study subjects: patients with knee osteoarthritis

Intervention: electroacupuncture, exercise therapy

Effect indicators: improvement in pain and functional indicators after 8 weeks of treatment

Study type: Randomized controlled trial

Research period : April 2022 to April 2024

**2. Protocol**

**Participants**

Diagnosis refers to the 1995 edition of the American College of Rheumatology (ACR) Guidelines :

①Knee pain most of the time in the past month ;

② X-ray shows osteophyte formation ;

③The synovial fluid examination is consistent with OA ;

④Age ≥ 40 years old;

⑤Morning stiffness ≤30min;

⑥There is bone friction sound.

who meet ① + ② or ① + ③ + ⑤+⑥ or ① + ④ + ⑤+⑥ can be diagnosed with KOA .

KL radiographic classification of knee osteoarthritis:

Level 0: normal;

Grade I: The joint space is suspiciously narrowed and there may be osteophytes ;

Grade II: There is obvious osteophyte and the joint space is suspiciously narrowed;

Grade III: moderate amount of osteophytes , clear joint space narrowing, and sclerotic changes;

Grade IV: A large number of osteophytes , significant narrowing of the joint space, severe sclerotic lesions and obvious deformity.

Inclusion criteria:

①Age between 45-75 years old, male or female;

② Unilateral / bilateral knee joint pain with duration of more than 3 months;

③ Radiological examination within 6 months shows KL grade II or III , and the medial compartment is severely involved ;

④ NRS ≥ 4 ;

⑤Sign the informed consent form

Exclusion criteria:

① Have a history of knee surgery or are waiting for knee surgery (knee replacement or knee arthroscopy);

② Knee pain caused by other diseases (such as loose bodies in the joint, severe effusion in the joint cavity, infection, malignant tumors, autoimmune diseases, trauma, fractures, gout, lumbosacral diseases, etc.);

③ Assess joints with a history of arthroscopy within 1 year and intra-articular injection in the past 6 months;

④ The knee joint has received acupuncture treatment/exercise therapy within the past 6 months;

⑤ Severe acute or chronic organic or psychiatric neurological diseases;

⑥ Coagulation disorders (such as hemophilia, etc.);

⑦ Have a pacemaker or epilepsy;

⑧ Women preparing for pregnancy, pregnancy and lactation;

⑨ Participated in other clinical studies within the past month;

**Randomization**

The trial adopted the stratified block randomization method, with the center as the stratification factor and the block length changing dynamically. Patients with knee osteoarthritis were randomly assigned to the electroacupuncture group and the exercise therapy group at a ratio of 1:1. Random sequences were generated by independent statisticians using SAS version 9.3 software programming. When there are qualified subjects, the clinical research coordinator (CRC) enters the patient information through the PAD to obtain the random number, thereby achieving allocation concealment and central randomization.

**Blinding**

The outcome evaluators and statistical analysts were blinded. Sports physicians, acupuncturists, and patients were not blinded.

**Intervention**

**(1) Electroacupuncture group**

If the patient has unilateral knee joint disease, the acupuncturist will perform acupuncture treatment on the affected knee joint, and the affected joint will be used as the evaluation joint during the test. If the patient has bilateral knee joint disease, the acupuncturist will perform acupuncture treatment on both knee joints. During the trial, the more severe side will be used as the joint for evaluation; if the conditions on both sides are similar, the joint will be selected by tossing a coin. Evaluate the joints.

The treatment is performed 3 times a week, and the needle is left in for 30 minutes for each treatment. The interval between each treatment is 1-2 days, for a total of 24 treatments in 8 weeks. When subjects need to take analgesics for knee joint pain , they can take emergency medication (diclofenac sodium) as needed . The subjects' medication time, dosage and symptom relief time must also be recorded .

Using a semi-standardized acupoint selection method, Dubi (ST35), Inner Knee Eye (EX-LE5), Ququan (LR8), Xiyangguan (GB33), and Ashi points (the most painful points perceived by the patient/doctor’s palpation and compression the most painful point) as the main acupoint, and then select 3 acupoints from the following acupoints according to the patient's disease syndrome . Allocation of acupoints along the meridian:

①Foot Yangming Meridian syndrome: Liangqiu (ST34) , Zusanli (ST36), Futu (ST32), Fenglong (ST40), Heding (EX-LE2);

② Foot Shaoyang Meridian Syndrome: Fengshi (GB31), Waiqiu (GB36), Yanglingquan (GB34), Xuanzhong (GB39), Foot Linqi ( GB41);

③ Full Taiyang Meridian Certificate: Weiyang (BL39), Weizhong (BL40), Chengshan (BL57), Kunlun BL60);

④Syndrome of three yin meridians of foot: Knee Guan (LR7), Yinlingquan (SP9), Sea of Blood (SP10), Yingu (KI10), Gongsun (SP4), Sanyinjiao (SP6), Taichong (LR3), Taixi (KI3).

Operation: Use disposable sterile acupuncture needles (produced by Suzhou Huatuo Medical Instrument Co., Ltd., 0.30mm×40mm or 0.30mm×50mm). The acupuncture site and the hands of the acupuncturist should be strictly disinfected with 75% alcohol, and the acupuncturist should puncture the needle. Transparent to the skin. Acupoint positioning is based on WHO international acupoint positioning standards.

After inserting the needle, perform acupuncture on all acupuncture points for about 10 seconds, depending on the degree of local soreness and swelling in the subject. After gaining Qi, select Ququan, Xianyangguan ( one pair of electrodes) and 2 matching points (another pair of electrodes) to connect. HANS-200A acupoint nerve stimulator (produced by Nanjing Jisheng Medical Technology Co., Ltd.) uses sparse and dense waves, frequency 2/100Hz, and the current intensity is based on patient comfort.

**(2) Exercise therapy group**

The training course focuses on neuromuscular training, and each training lasts 90-120 minutes, lasting 8 weeks, twice a week, with a total of 16 courses. The first two courses are education for patients. The first education course includes the definition, clinical manifestations, diagnostic methods, causes, risk factors and anatomy knowledge of knee joint, etc., aiming to help patients increase their understanding of knee osteoarthritis. Understanding of the disease, the second course introduces patients to the main treatment methods, preventive measures and pain management of knee osteoarthritis, and introduces the role of exercise therapy in the treatment of knee osteoarthritis. Each training session is then divided into three parts: warm-up exercise, neuromuscular training, and cool-down exercise.

Warm-up exercise: lasts about 10 minutes , aiming to improve the efficiency and safety of subsequent exercise .

Neuromuscular training: The main part of each course includes core stability training, muscle strength training, dynamic posture control and daily functional training, focusing on "closed chain exercise". The training is divided into 5 groups, with 2 movements in each group: pelvic lift and sit-ups; knee bend and knee extension exercises; front and back and sideways sliding; hip abduction and adduction; chair sit-ups and step exercises. Perform each action for 3 cycles x 10 times.

Cool down exercise: including slow walking and lower limb muscle stretching training.

Training courses are conducted in groups of 8-10 people. During the training process, the therapist adjusts the intensity and difficulty of the training based on the completion of the patient's movements and the patient's pain feedback after training. At the same time, if the subjects are overweight, they are encouraged to lose weight, with the goal of losing 5KG within 6 weeks.

When subjects need to take analgesics for knee joint pain , they can take emergency medication (diclofenac sodium) as needed . The subjects' medication time, dosage and symptom relief time must also be recorded.

**Outcome Measures**

**Primary outcone**

The main outcome measure includes two dimensions. The primary outcome measure for pain is the change in Numerical Rating Scale (NRS) from baseline at week 8. The main outcome measure for function is the change from baseline in KOOS ADL.

**Secondary outcome**

**(1) Other self-reported outcomes of patients**

①KOOS Pain : Pain level score, there are 9 scoring items ( 0-4 points), the total converted score is from 0 to 100 , the lower the score, the higher the pain level.

②KOOS QOL : Quality of life score. There are 4 scoring items ( 0-4 points). The total converted score ranges from 0 to 100. The lower the score , the lower the quality of life.

④Patient’s global assessment

⑤Credibility and Expectation Questionnaire

**(2) Objective outcomes of body functions:**

① 30 -second sit-up test: The participant sits on a chair and places his feet flat on the floor, shoulder width apart, with his knees bent slightly more than 90 degrees. Cross your arms at the wrists, close to your chest. Measure the number of times within 30 seconds that the participant stood up completely, with their hips and knees fully extended, and then sat down completely , with their buttocks fully touching the seat . Depending on age and gender, there are different average rep interval standards. Compare the measured times with the average rep interval to evaluate objective physical function.

② 40-meter brisk walking test: Participants are asked to walk as fast as possible along a 10-meter walkway without running, then make a circle around a cone, then return, and repeat the walk for 40 meters , and calculate the completion time and average speed. Depending on age and gender, there are different average time or speed standards, and participants' values are compared with the standard to evaluate objective physical function.

③Physical activity level measured by pedometer: Use a bracelet-type pedometer to measure the subjects' average daily steps within 1 week.

Evaluation time points for the above secondary outcome indicators: before the first treatment ( week 0) , week 4 ± 1 day, week 8 ± 3 days, week 16 ± 3 days, week 24 ± 3 days

**(4) Safety results**

Observe the safety of the two therapies through adverse event evaluation, and determine whether the adverse events are related to acupuncture/exercise therapy by DSMB

Evaluation time point: throughout the trial period

**Sample size**

Based on our preliminary trial data, we speculated that the inter-group difference in effect size between the exercise treatment group and the acupuncture treatment group would not exceed 0.5 standard deviation. At a two-sided α = 0.025 (adjusted for both outcomes) , 77 participants per treatment group were needed to test with 80% confidence an effect size difference of 0.5 (medium effect size). Considering a 20% dropout rate, it is estimated that a total of 196 subjects will need to be recruited.

**Statistical method processing**

1. Use SPSS 26.0 software for statistical processing. For all statistical tests, P < 0.05 will be considered as statistically significant differences. Measurement data are expressed as mean ± standard deviation or median (25th percentile, 75th percentile), and count data are expressed as percentages.

2. Balance analysis of basic values: Use analysis of variance or χ2 ^test^ to compare demographic information and other basic value indicators to measure the balance of the two groups.

3. All randomly grouped cases will be included in intention -to-treat analysis (ITT ); missing data will be filled in using multiple imputation.

4. Per-Protocol Population was also used for the primary outcome. The acupuncture group was defined as per -protocol as having received at least 20 acupuncture treatments without any obvious violation of the protocol; the exercise therapy group was defined as per-protocol as having received at least 12 acupuncture treatments. .

5. Measurement data uses t test, and count data uses χ2 ^test^ or non-parametric test.

**Dropping and handling of cases**

Dropout criteria: For some reasons, although the subject passed the informed consent and passed the screening to obtain a random number, he was unable to complete the treatment course and observation period specified in the research plan, and was regarded as a dropout case.

Handling of dropout cases: When a subject drops out, the researcher needs to contact the subject as much as possible through door-to-door visits, follow-up appointments, phone calls, letters, etc., ask for the reasons, record the time of the last acupuncture, and complete what can be accomplished. assessment items. Relevant trial data should be properly preserved for dropout cases, both for archiving purposes and for statistical processing of intention-to-treat analysis. Patients who have lost it do not need to replace it.

**Termination criteria**

① The patient experienced serious adverse reactions, and the clinical trial in this case needed to be stopped based on the doctor's judgment.

② If other diseases and syndromes that affect trial observation occur and the clinical trial should be stopped according to the doctor's judgment, they will be treated as invalid cases.

③ Important deviations occurred during the implementation of the clinical trial plan, such as poor compliance , making it difficult to evaluate the efficacy.

④ Subjects who are unwilling to continue the clinical trial during the clinical trial process and request the supervisor to withdraw from the clinical trial.

*ethical principles*

The design and implementation of any clinical trial, while ensuring scientific validity, must comply with ethical requirements and protect the health and rights of subjects who are vulnerable groups.

1) Ethical review of the project This project is a randomized controlled clinical study. Before the trial is implemented, the principal investigator should submit the trial protocol, informed consent form, subject recruitment materials, CRF, researcher resume, and other ethics committee explanations of the project review decision to the ethics committee of the clinical trial unit for review and approval. . After review and approval, if important modifications need to be made to the trial protocol, it should be submitted again to the ethics committee for review and approval.

2) Risk-benefit assessment Subjects and society will likely benefit from this study, including: ① The patient's condition may be improved. ② During the trial, the patient's condition will be comprehensively evaluated, and the patient will receive careful and thoughtful medical services. ③ May benefit similar patient groups. Participating in this trial may face risks, including: ① The risk of the disease itself, such as aggravation of the condition and the occurrence of complications; ② The risk of potential drug damage and adverse reactions, such as dizziness. In order to cope with risks and successfully complete this study, the following measures will be taken: ① The trial protocol will be entrusted to a professional team with rich clinical experience and drug evaluation to design, and follow the principles of minimizing risks, discomfort, and pain. ②Refine the conditions for researchers to decide to withdraw, and rationally design combined medication regulations to protect the health and interests of subjects.

research project to interested parties . Recruitment advertisements must include at least the following content and must be submitted to the Ethics Committee for review: ① Name and address of the researcher and research institution; ② Research conditions or research purpose; ③ Overview of inclusion and exclusion criteria; ④ Main benefits ; ⑤ Trial plan and time; ⑥Contact information.

*Medical measures after the trial*

If adverse events occur after the trial, corresponding treatment will be provided, but no other medical measures will be provided.

*Plan modification*

Modifications to this clinical trial protocol and informed consent form must be reviewed and approved by the ethics committee.
