# Supplementary material for: Electroacupuncture versus exercise in patients with knee osteoarthritis: Study protocol for a randomized controlled trial
Source: PLoS One. 2024 Jun 11;19(6):e0305105. doi: 10.1371/journal.pone.0305105 (PMC11166276; doi:10.1371/journal.pone.0305105)
Supplement: S3 File — (DOCX) [file pone.0305105.s003.docx]

**Model consent form**

**Informed Consent Form • Informed Notice Page (Translated Version)**

Dear Madam/Sir:

You are invited to participate in a clinical study titled "Electroacupuncture versus Exercise in Patients with Knee Osteoarthritis: A Randomized Controlled Trial." The study aims to evaluate the effectiveness and safety of electroacupuncture compared to exercise therapy in treating knee osteoarthritis.

Before you decide whether to participate in this study, please carefully read the following information. It will help you understand the purpose of the study, its procedures and duration, as well as the potential benefits, risks, and discomforts associated with participation. If you wish, you may discuss this with your family or friends, or seek explanations from your doctor to assist you in making your decision.

**Introduction**

1. We will conduct the " Electroacupuncture versus Exercise in Patients with Knee Osteoarthritis: A Randomized Controlled Trial." The principal investigator of this project is Professor Cunzhi, Liu, Dean of the School of Acupuncture-Moxibustion and Tuina, Beijing University of Chinese Medicine.

2. This is a clinical trial.

3. Knee osteoarthritis, also known as degenerative knee joint disease or hypertrophic knee arthritis, is one of the common joint diseases in clinical practice, often occurring in middle-aged and elderly individuals, especially females. The prevalence of knee osteoarthritis is approximately 8.1% in China. Its high incidence and disability rates not only impose a heavy economic burden on patients but also significantly affect their quality of life. Currently, the treatment of knee osteoarthritis mainly involves symptomatic treatment for mild to moderate cases and surgical measures for severe cases. Although drug therapy for knee osteoarthritis has shown promising short-term efficacy, relapse often occurs with prolonged cessation of medication, and many patients experience varying degrees of adverse reactions. Electroacupuncture therapy, with its long history in the treatment of knee osteoarthritis, is widely accepted by patients due to its high safety. Exercise therapy has been advocated by many countries as an effective treatment for knee osteoarthritis, and various degrees of implementation have been carried out both domestically and internationally. It is urgent to further validate the differences between electroacupuncture and exercise therapy in improving different symptoms of knee osteoarthritis, as well as the differences in populations suitable for each therapy.

4. This study will include 196 patients with knee osteoarthritis. The study will last for approximately 24 weeks. Before enrollment in the study, the researcher will refer to the inclusion and exclusion criteria, inquire about your relevant symptoms, and inform you of the details of the study, addressing all related issues. If you enter the trial, you will have an equal chance of being assigned to either the electroacupuncture group or the exercise therapy group and receive the corresponding treatment. If assigned to the electroacupuncture group, you will receive electroacupuncture treatment three times a week for 8 weeks; if assigned to the exercise therapy group, you will receive exercise therapy twice a week for 8 weeks. Regardless of the group you are assigned to, visits and relevant scale assessments will be conducted at approximately week 4, week 8, week 16, and week 24. These assessments will include evaluation of the impact of electroacupuncture on pain and function in patients using questioners such as the Numerical Rating Scale and Knee Injury and Osteoarthritis Outcome Score, objective assessment of physical function using the 30-Second Chair Stand Test, 40-Meter Fast-Paced Walk Test, and pedometer-measured physical activity levels, and observation of safety of electroacupuncture and exercise therapy through assessment of adverse events. Throughout the trial, you may receive diclofenac sodium as a temporary oral analgesic when experiencing unbearable pain. Please keep all packaging and remaining medication, as the researcher will collect these materials for documentation at the end of the trial.

5. Inclusion criteria

You will be eligible if you: aged 45-75, any gender; meet the American College of Rheumatology (ACR) diagnostic criteria of KOA; have knee pain for more than 3 months; have radiological examination within 6 months indicating II or III in Kellgren-Lawrence grade with medial more than lateral tibiofemoral osteoarthritis in at least one knee; score 4 or greater in numerical rating scale (NRS) during flat walking for the past week; are willing to sign the informed consent.

6. Exclusion criteria
You will be ineligible if meet the following points: history of knee surgery for the evaluating knee or waiting for any surgery for either knee; knee pain due to other diseases (eg meniscus tear, rheumatoid arthritis, joint cavity infection, malignancy, gout, lumbosacral diseases with symptom of lower extremity, etc. arthroscopy history within 1 year or intraarticular injection history within 6 months for the evaluating knee; either knee received acupuncture/exercise therapy for the last 6 months; severe acute or chronic organic or neuropsychiatric disorder; disorders of coagulopathy (eg hemophilia); pacemakers or epilepsy; pregnancy preparation, pregnant or lactation period; participated in other clinical studies within the past 1 month.

7. Withdrawal Criteria:

You can withdraw from the study if:

(1) Inadequate efficacy is observed;

(2) Intolerance to adverse reactions occurs;

(3) You wish to pursue alternative treatment methods;

(4) Or withdraw from the trial without providing any reason.

8. Termination Criteria:

Your intervention may be terminated if:

(1) Significant abnormalities in vital organ function occur;

(2) Drug allergic reactions are observed;

(3) Poor compliance is noted;

(4) Deterioration of the condition or occurrence of serious adverse reactions necessitates cessation of trial treatment;

9. Post Exclusion Criteria:

You may be excluded from the study if:

(1) You violate the inclusion and exclusion criteria to enter the trial;

(2) You use concomitant medication not specified in the protocol;

(3) You are assigned to the wrong treatment group;

(4) Extremely poor treatment compliance.

10. Study Suspension/Termination Criteria:

The study may be suspended or terminated if:

(1) Effective assurance of subject safety cannot be maintained;

(2) The sponsor fails to submit required safety updates during the research and development period;

(3) The sponsor fails to promptly address and report suspected and unexpected serious adverse reactions;

(4) Evidence indicates that the study intervention is ineffective;

(5) Falsification occurs during the course of the clinical trial;

(6) Other violations of the quality management standards for clinical trials occur. When a significant, unexpected serious adverse reaction occurs during the clinical trial, or when evidence indicates serious quality issues with the clinical trial, the sponsor should immediately suspend the clinical trial. Relevant departments may, according to their responsibilities, order adjustments to the clinical trial protocol, suspend, or terminate the clinical trial.

**Explanation of Potential Benefits of Participating in the Study**

Benefits to the Social Community:

We hope that the information obtained from your participation in this study will benefit patients with similar conditions in the future.

Benefits to the Participants Themselves:

Research indicates that both electroacupuncture and exercise therapy can provide pain relief and improve symptoms associated with knee osteoarthritis. This study involves receiving electroacupuncture three times a week or exercise therapy twice a week for the treatment of knee osteoarthritis, which may improve symptoms such as knee pain and functional impairment, thus enhancing your quality of life.

**Explanation of Potential Discomforts and Risks to Participants**

Discomfort caused by the use of investigational drugs or devices:

Adverse reactions may occur during the trial. Previous clinical studies have shown that electroacupuncture and exercise therapy have not resulted in serious adverse reactions. Common adverse events associated with electroacupuncture may include mild events such as bruising, fainting, needle retention, and soreness after treatment, while those associated with exercise therapy may include pain and spasms. If any of these occur, treatment will be paused until the adverse reaction subsides, and a decision will be made whether to continue treatment.

Risks associated with participation in the trial itself:

When considering whether to participate in this study, please carefully consider the potential impact of treatment and follow-up on your daily work, family life, etc. Consider the time and transportation issues for each treatment session and follow-up visit. If you have any questions about the content of the trial, feel free to consult us. For your safety and to ensure the validity of the study results, you will not be allowed to participate in any other clinical trials related to acupuncture, exercise, drugs, and medical devices during the study period.

**Alternatives to Participation in the Study**

You may choose not to participate in this study, which will not have any adverse effects on your access to conventional treatment. Currently, conventional treatment methods for your health condition include non-steroidal anti-inflammatory drugs, glucosamine, intra-articular injections, or arthroscopic surgery. In severe cases, joint replacement may be an option.

**Instructions on Concomitant Medications and Restrictive Treatments**

During the trial, please refrain from undergoing any other treatments that may affect the symptoms of knee osteoarthritis, including drug therapy (non-steroidal anti-inflammatory drugs, opioid drugs, etc.) and other physical therapies (such as massage, hydrotherapy, etc.). If you experience unbearable pain during the trial, you may request diclofenac sodium sustained-release tablets (Beijing Novartis Pharmaceutical Co., Ltd.) 150mg as emergency medication. The recommended dosage is 75mg once daily, as per the medication instructions.

**Related Expenses of Participating in the Study**

The cost of 24 sessions of electroacupuncture treatment or 16 sessions of exercise therapy, as well as the as-needed use of diclofenac sodium, will be covered by the research institution.

**Provisions for Compensation and Compensation**

When your health is harmed during your participation in this study, please inform the investigator (Liu Cunzhi, Phone number: xxxxxxxxxxx), and necessary medical measures will be taken. Compensation will be provided according to the degree of damage in accordance with relevant national laws and regulations.

**Confidentiality of the Study**

All information related to you, including your identity, medical history, condition, physical examinations, and laboratory test results, will be kept strictly confidential within the limits permitted by law. Investigators, authorized inspectors appointed by the sponsor, ethics committees, and the National Medical Products Administration are allowed to access your medical records related to this study to verify the authenticity and accuracy of the data collected in this study, without involving your personal details. Your name will not appear in any public documents or reports related to this study.

**Clarification of Participants' Rights**

Your participation in the study is entirely voluntary, and you have the right to withdraw from the study at any stage without penalty or loss of benefits, and it will not affect the treatment provided by your doctor. If you decide not to participate in this study or withdraw from the study at any time after it starts, please contact your doctor promptly.

**Handling of Participant Complaints**

If you have any complaints during the study, please contact the principal investigator (Phone number: xxxxxxxxx) or the office of the ethics committee (Phone number: xxxxxxxx).

**Informed Consent Form • Consent Signature Page**

**Participant**

I have carefully read the " Electroacupuncture versus Exercise in Patients with Knee Osteoarthritis: A Randomized Controlled Trial" and fully understand the purpose, content, methods, as well as the potential benefits and risks of participating in the clinical study. The researcher has provided clear explanations of relevant medical terms, and all questions I asked have been answered in a clear and understandable manner. I understand that I can refuse to join the study or withdraw from it at any time and under any circumstances, without affecting my medical treatment or rights. My participation in this study is entirely voluntary, and I have given it full consideration. I understand the therapeutic effects and potential risks that the study interventions may have on my condition, and I have obtained comprehensive and truthful information related to this study. I fully understand and support this clinical research. In the absence of any pressure and with the freedom to choose, I voluntarily participate in this clinical study and agree to cooperate with the study doctor, adhere to the prescribed medication, undergo examinations as required, and complete this clinical study. I agree that when necessary, personnel from the National Medical Products Administration, clinical research inspectors, and monitors may review my medical records and research data. I will receive a copy of the informed consent form signed and dated.

Patient (Signature): _______________ Date: ____ Year ____ Month ____ Day___

(Or Legal Guardian (Signature):______) Relationship with Patient: ____________

Phone Number: __________________

**Researcher**

I confirm that I have provided detailed explanations of the content, procedures, potential risks, and benefits of this study to the participant mentioned above. I have answered any questions raised by the patient satisfactorily, and the patient has expressed understanding and satisfaction with the responses provided.

Researcher (Signature): _____________ Date: ____ Year ____ Month ____ Day___

Phone Number: __________________
