# Supplementary material for: Electroacupuncture versus exercise in patients with knee osteoarthritis: Study protocol for a randomized controlled trial
Source: PLoS One. 2024 Jun 11;19(6):e0305105. doi: 10.1371/journal.pone.0305105 (PMC11166276; doi:10.1371/journal.pone.0305105)
Supplement: S4 File — (DOCX) [file pone.0305105.s004.docx]

**Checklist for Protocols According to the TIDieR Guideline**

| Brief name | Exercise therapy | Electroacupuncture |
| --- | --- | --- |
| Why | Both exercise therapy and electroacupuncture were proved to relieve pain and dysfunction in managing knee osteoarthritis. It is not clear about the differences between the 2 therapies in effect. | |
| What materials | Stationary bike, fitness ball, rubber band, sliding plate, simulated step and bar bell will be used during exercise as required by the movement. | Disposable sterile needle and electroacupuncture therapeutic apparatus. |
| **What procedures** | Part 1. Warming up:  This part involves 10 minutes of ergometer cycling. The workload will be set individually to improve the efficiency and safety during subsequent exercise.  Part 2. Circuit program:  Circuit 1: Core stability/postural function  A. Pelvic-lift: lift the pelvis with both feet on the exercise ball; B. Sit-ups: curl the abdomen with both feet on the exercise ball.  Circuit 2: Postural orientation  A. Slide-exercise forward-backward: slide back and forth on the sliding plate with one foot; B. Slide-exercise sideways: slide sideways on the sliding plate with one foot.  Circuit 3: Lower extremity muscle strength  A. Hip abductors/hip adductors: Standing, abduct or adduct the hip with resistance of a rubber band; B. Knee extensors/knee flexors: Sit in a chair, extend and flex the knee under the resistance of a rubber band.  Circuit 4: Functional exercises  A. Chair stands: Sit in a chair, stand up and sit down at a constant pace; B. Stair climbing: Standing in front of the simulated step, step-up and step-down.  Part 3. Cooling down  This part consists of walking, mobility exercises for the lower extremities and stretching exercises for the lower extremity muscles. | Five fixed acupoints and 3 optional acupoints will be inserted. After the needle inserted, lift, thrust and thrill smoothly for at least 10s to achieve De qi. Two electric lines will be connected: LR8 and GB33, and 2 of 3 adjunct acupoints. |
| **Who provided** | By 3 licensed physiotherapists with more than 3 years of clinical experience. All physiotherapists were trained in standardized operating procedures prior to the start of the study. | By 3 licensed acupuncturists with more than 3 years of clinical experience. All acupuncturists were trained in standardized operating procedures prior to the start of the study. |
| How | Delivered by supervised in groups of 8-10 patients. | Delivered in separate therapeutic beds. |
| Where | In a GLA:D authorized clinic (Wentao Clinical Exercise) | In the acupuncture department of the study center. |
| **When and how much** | Twice a week for 8 weeks, with each session lasting approximately 60 minutes. | Three times a week for 8 weeks, with each session lasting approximately 40 minutes. |
| **Tailoring** | Each movement in the circuit exercise program will be stratified into 3 levels. Each level increases in strength or amplitude. Tailor the load intensity of each movement according to patient's ability to complete the movement and their pain feedback. | Tailor the optional acupoints according to the symptoms and acupuncture theory. |
| **How well (planned)** | Both physiotherapists and acupuncturists will receive prior training in how to deliver the intervention. The inspectors will evaluate the study every 2 months. Compliance with both interventions will be recorded. | |

Pictures or videos of the exercise therapy could be available at the GLA:D® official link: https://gladinternational.org/glad-hip-and-knee/.
